# The *flipflop* orphan genes are required for limb bud eversion in the *Tribolium* embryo

**DOI:** 10.1186/s12983-017-0234-9

**Published:** 2017-10-19

**Authors:** Susanne Thümecke, Anke Beermann, Martin Klingler, Reinhard Schröder

**Affiliations:** 1 Institut für Biowissenschaften, Universität Rostock, Albert-Einsteinstr 3, D-18059 Rostock, Germany; 2 Universität Tübingen, Auf der Morgenstelle 15, D-72076 Tübingen, Germany; 3 Friedrich-Alexander-Universität Erlangen-Nürnberg, Department Biologie Abt. Entwicklungsbiologie, Staudtstr. 5, D-91058 Erlangen, Germany

**Keywords:** Appendage formation, Epithelial morphogenesis, Evagination, Orphan *flipflop* gene, PCP, RhoGEF2, Tissue folding, *Tribolium castaneum*

## Abstract

**Background:**

Unlike *Drosophila* but similar to other arthropod and vertebrate embryos, the flour beetle *Tribolium castaneum* develops everted limb buds during embryogenesis. However, the molecular processes directing the evagination of epithelia are only poorly understood.

**Results:**

Here we show that the newly discovered genes *Tc-flipflop1* and *Tc-flipflop2* are involved in regulating the directional budding of appendages. RNAi-knockdown of *Tc-flipflop* results in a variety of phenotypic traits. Most prominently, embryonic limb buds frequently grow inwards rather than out, leading to the development of inverted appendages inside the larval body. Moreover, affected embryos display dorsal closure defects. The *Tc-flipflop* genes are evolutionarily non-conserved, and their molecular function is not evident. We further found that *Tc-RhoGEF2*, a highly-conserved gene known to be involved in actomyosin-dependent cell movement and cell shape changes, shows a *Tc-flipflop*-like RNAi-phenotype.

**Conclusions:**

The similarity of the inverted appendage phenotype in both the *flipflop*- and the *RhoGEF2* RNAi gene knockdown led us to conclude that the *Tc-flipflop* orphan genes act in a Rho-dependent pathway that is essential for the early morphogenesis of polarised epithelial movements. Our work describes one of the few examples of an orphan gene playing a crucial role in an important developmental process.

**Electronic supplementary material:**

The online version of this article (10.1186/s12983-017-0234-9) contains supplementary material, which is available to authorized users.

## Background

The general bauplan of the insect leg is highly conserved in evolution and so are the genes controlling appendage growth and patterning [2]. Yet, principal topological differences within the insects exist. In the fruit fly *Drosophila*, the leg anlagen invaginate from the larval epidermis and internalise to develop inside the body cavity as imaginal discs. During pupation, appendages evert and only become functional in the adult. In contrast, ventral appendages of the flour beetle *Tribolium* start as everting epidermal bulges that subsequently grow in length during embryogenesis. This mechanism of appendage formation is representative for most arthropods and similar to apical epidermal ridge formation in vertebrates [59]. Eventually *Tribolium* larvae hatch with fully differentiated, functional appendages [49].

Bud formation takes place in a restricted area of the epithelium where cells collectively polarise, undergo cell shape changes and, as a consequence, evaginate. Once this crucial decision is made, the bud grows in length and eventually differentiates [19, 54, 60]. The coordinated contractility of a group of cells at their apical or basal cortices provides the cellular basis for this morphogenetic event: apical constriction leads to tissue invagination while basal constriction results in the formation of an external bud. Constriction at one cortex of a cell usually goes along with expansion of the membrane at the opposite side [22].

To date, morphogenetic processes that involve apical constriction are intensely studied in a variety of developmental contexts. Most prominent examples are the infolding of cell sheets during gastrulation or neurulation, blastopore formation, trachea development, dorsal- and neural tube closure as well as embryonic tissue sealing during wound healing [22, 25, 27, 34, 40, 45].

However, tissue eversion as a consequence of basal constriction is less well understood and has been analysed in only a few cases: the formation of the midbrain-hindbrain boundary constriction and morphogenesis of the optic-cup in vertebrates, and notochord formation in an urochordate [9, 14, 28, 29, 33]. Classical studies in the polyp *Hydra* describe basally constricted cells within epithelial sheet curvature during reproductive bud initiation [12, 58].

Different cellular mechanisms such as differential growth or compressing forces from neighbouring cells have also been shown to initiate tissue bending and have been described for morphogenetic events like branching of developing epithelia or gut looping [56]. Moreover, all the described processes are likely to synergise with other types of cell behaviour, such as directed cell migration into the region where a bud will form, or changes in adhesive properties once a bud protrudes out of the plane of an epithelium.

In any case, epithelial cell shape changes require the dynamic and spatial reorganisation of the actomyosin network. Its assembly and disassembly is controlled by small GTPases like RhoA (ras homologue family member A). RhoA becomes activated by the guanine nucleotide exchange factor RhoGEF2 which is transported to the apical cell cortex along the polarised microtubule network through association with the plus-end binding protein EB1 at the tips of the growing microtubules. At the apical cortex, active RhoA triggers myosin contraction through the Rho-associated coiled-coil kinase (ROCK) [27, 41].

Rho family GTPases, the effectors of myosin constriction, are also a target of the planar cell polarity (PCP) signalling pathway [30] that coordinates the behaviour of cells within an epithelium. The aligning of activated myosin through PCP along an axis eventually leads to polarised tissue-bending and -folding in one direction exemplarily seen during neural tube folding [35]. PCP involves the non-canonical Wnt-signaling pathway upstream of Rho [6].

In addition, the Rho/RhoGEF2/ROCK cassette is employed by other upstream signals and factors like the Jun-N-terminale-kinase (JNK-) or G-protein-coupled receptors (GPCR) [17, 21, 37].

In order to further understand the crucial aspects of early evagination processes we analysed genes in *Tribolium* resulting in a hitherto not described larval knockdown phenotype of inverted rather than everted larval appendages.

Here, we focus on the novel *Tc-flipflop1* (*Tc-ff1*) gene that was identified in the genome-wide RNAi (RNA interference) screen iBeetle [48] and a newly identified *flipflop* paralogue (*Tc-ff2*). RNAi-based knockdown of *Tc-ff* results in the formation of inverted but otherwise fully developed legs inside the larval thorax rather than growing out distally. A similar appendage phenotype was observed in an insertional mutant identified in the GEKU screen [55] which is located in the *RhoGEF2* gene. Furthermore, we found that both *Tc-flipflop* genes as well as *Tc-RhoGEF2* are essential for the integrity of morphogenetic movements of embryonic cells and extraembryonic membranes. We propose that in the limb-field, the very early decision of an epithelium to either invaginate or evaginate depends on Rho associated signalling with the novel *Tc-ff* genes as essential mediators to secure tissue eversion. Whether restricted only to *Tribolium* or fast evolving yet present in other animals, the *Tc-ff* orphan genes highlight the involvement/importance of novel factors in early epithelial morphogenesis and appendage formation.

## Methods

### Animal stocks

Beetle adults and embryos (*Tribolium castaneum*, nGFP line) [43] were kept under standard conditions on wheat flour at 30 °C [5] and used for parental RNAi, *in situ* hybridisation and live imaging.

### RNAi mediated knockdown

For gene specific knockdown non-overlapping fragments were ordered from Eupheria Biotech GmbH (1 μg/μl, 3 μg/μl). For parental RNAi young adult females were sedated on ice and fixed on a petri dish using double-sided adhesive tape. dsRNA (*Tc-ff*, 500 ng/μl; *Tc-RhoGEF2*, 200 ng/μl) was injected into the abdomen under a stereomicroscope using a glass capillary connected to a manually controlled syringe. Gene specific effects for all results shown were validated with at least two non-overlapping fragments (NOFs) for each gene (*Tc-ff1*: NOF1 basepairs 1–320 (xx-90,314-2), NOF2 bp 340–659 (xx-90,314-1), *Tc-ff2*: NOF1 bp 129–416 (xx-90,313-3), NOF2 bp 417–710 (xx-90,313-2), *Tc-RhoGEF2* NOF1 bp 2338–2664 (iB_03492), NOF2 bp 7408–7907) (iB_00510)). Eggs were collected and either fixed (in situ hybridisation, antibody staining, morphological analysis) or incubated at 30 °C to develop a cuticle. *Tc-Dll* dsRNA was injected as a control to validate gene-specific effects.

### Molecular biology and expression analysis

For whole mount *in situ* hybridisation gene-specific primers were used (Metabion) to amplify gene fragments via PCR using cDNA synthesised from total RNA. The amplified fragments were subcloned into the pCR4 vector (TOPO-TA Cloning Kit, Invitrogen). In vitro transcription for synthesis of DIG-labelled RNA probes was performed using the DIG RNA Labelling Kit (Roche Applied Science). Whole mount in situ hybridisation was performed as previously described [46]. Staining was achieved through application of an Anti-Digoxigenin-AP antibody in combination with NBT/BCIP (Roche Applied Science).

Antibody staining was carried out as previously described [51] using antibodies raised against short peptides corresponding to Flipflop1 (NH2-CPKTTKPKAK-CONH2), Flipflop2 (NH2-CSKNTEHKTK-CONH2) (Pineda Antibody-Service) and Cleaved Dcp-1 (#9578, Cell Signaling Technology), respectively. A Biotin-SP-conjugated AffiniPure Anti-Rabbit lgG antibody was used as secondary antibody (Jackson ImmunoResearch Europe Ltd). Staining was carried out using Vectastain ABC-AP (Vector Laboratories) and NBT/BCIP.

### Live imaging

Eggs (nGFP) were dechorionated in diluted bleach, washed, positioned on a microscope slide and covered with halocarbon oil (Voltalef 10S). Injections were performed under an inverted microscope using a micromanipulator. Live imaging was carried out using a Zeiss Z.1 microscope (Zen 2.3 software) equipped with a motorised stage. Pictures were taken every 1–5 min. Images and videos were processed with Adobe Photoshop CS5.

### Databases

#### Genomes:

*Tribolium castaneum*: http://bioinf.uni-greifswald.de/gb2/gbrowse/tcas4/

UCSC Genome Browser: https://genome-euro.ucsc.edu/cgi-bin/hgHubConnect? redirect=manual&source= genome.ucsc.edu

#### RNAi induced phenotypes:

iBeetle-Base: http://ibeetle-base.uni-goettingen.de [10].

#### Protein structure:

HHpred: https://toolkit.tuebingen.mpg.de/#/tools/hhpred [52].

SMART: http://smart.embl-heidelberg.de/smart/set_mode.cgi?NORMAL=1 [50].

## Results

### The novel gene *Tc-flipflop* reveals a new RNAi knockdown phenotype

**Fig. 1:**
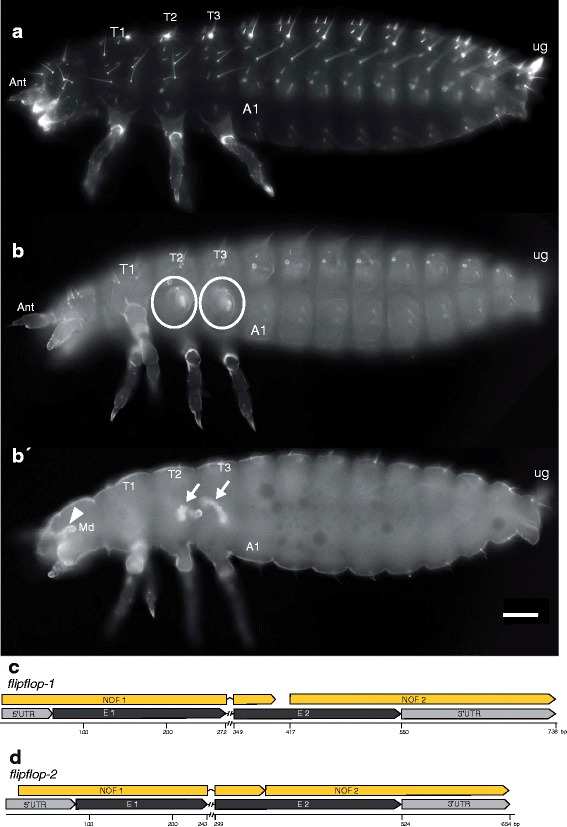
Larval *Tc-flipflop* ^RNAi^ phenotype. (**a**) Wildtype cuticle. All appendages develop as everted structures. Each thoracic segment bears one pair of visible legs. (**b**-**b′**) *Tc-ff* ^RNAi^ phenotype. (**b**) Surface view, (**b´**) optical section. (**b**) In the thoracic segments 2 and 3, one leg is everted normally. Of the other legs, only the coxa is visible as an outer structure (circle) while the remaining leg is internalised (**b′**, arrows). (**c**, **d**) Gene organisation of *Tc-ff1* (**c**) and *Tc-ff2* (**d**) genes including NOF (non-overlapping-fragment) positions used for RNAi experiments. A1 abdominal segment 1; Ant antenna; Md mandible; T1–3 thoracic segment 1–3; ug urogomphi; scale bar (**a**-**b′**) 100 μm; all panels in all pictures: anterior to the left

**Fig. 2:**
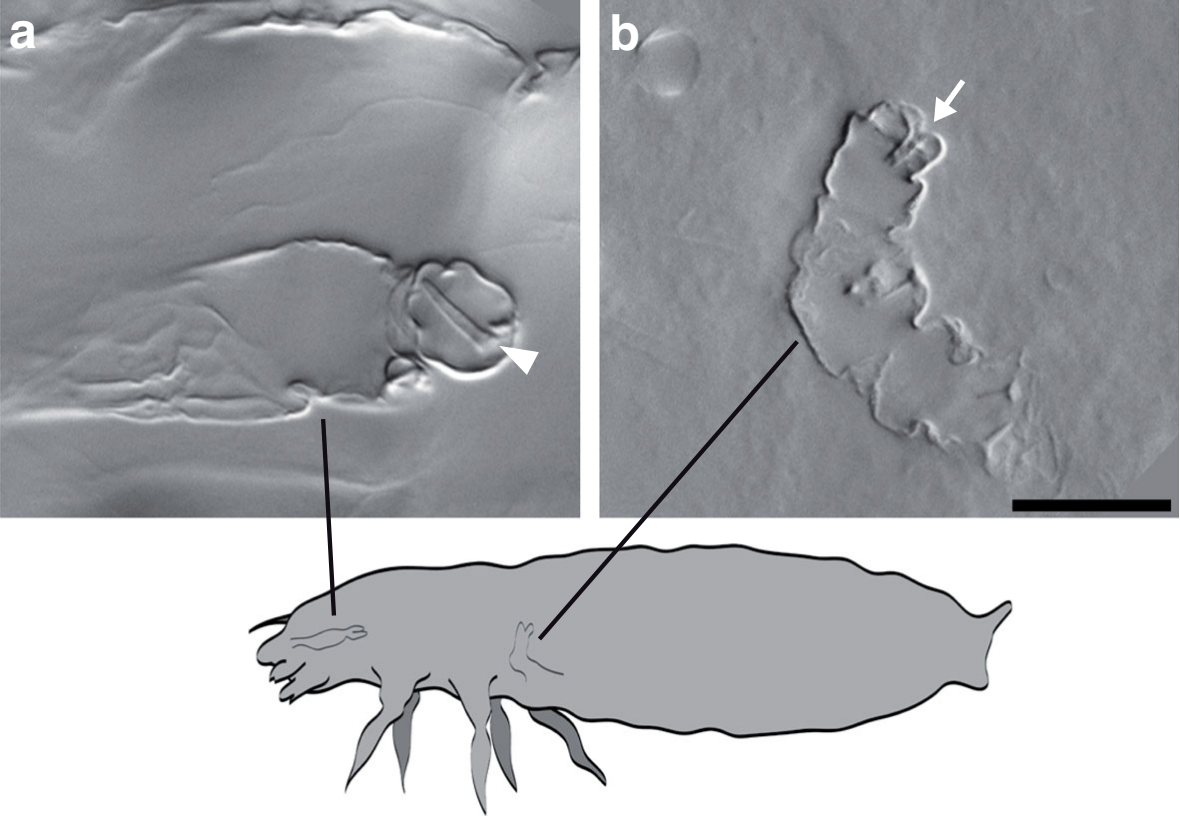
Detailed view of the larval *Tc-flipflop* ^RNAi^ phenotype. **a**, **b** Magnified view of a larval inverted antenna **a** and leg **b**. Internalised parts are fully differentiated; the distalmost structures (antennal flagellum) (arrowhead in a) and the pretarsal claw (arrow in b) develop normally and display the inside-out event. Scalebar 20 μm

In wildtype *Tribolium* larvae, appendages develop as everted structures outside the larval body (Fig. 1a). The RNAi mediated knockdown of the gene *Tc-flipflop1* (*Tc-ff1*) results in an enigmatic novel RNAi cuticle phenotype where larval appendages developed inside the larval body (outside-in phenotype). This gene was uncovered during the iBeetle prescreen when randomly selected cDNAs were tested for their function [48]. In *Tc-ff* ^RNAi^ cuticles, various numbers of head – or thoracic appendages appear to be missing or shortened when focussing onto the cuticle surface (Fig. 1b, circles). However, those appendages are indeed present inside the larva as seen in optical sections (Fig. 1b'). These inverted structures do not show any obvious developmental defects, revealing distal segments (flagellum, pretarsal claw) oriented towards the lumen, while podomers and joints seem fully differentiated (Fig. 2a, b).

### There are two *Tc-flipflop* genes in the *Tribolium* genome

In addition to the original *Tc-flipflop* gene, we identified an obvious *Tc-ff* paralog in the *Tribolium* genome. Both genes show 47% sequence similarity at the amino acid level and were therefore named *Tc-ff1* (*TC032552*) and *Tc-ff2* (*TC030881*), respectively. *Tc-ff1* and *Tc-ff2* are short genes (738 and 756 bp) with two exons each (Fig. 1c, d) that lack any conserved domains.

*ff1* is also present in other *Tribolium* species including *T. confusum*, *T. madens* and *T. freemani*. Due to their incomplete annotation, it is not yet clear whether those genomes all contain a true *ff2* paralogue (UCSC Genome Browser data, not shown). However, the genes cannot be found in any other sequenced genomes, including more distantly related coleopteran species.

In *Tribolium castaneum*, both *Tc-ff* genes are ubiquitously expressed in all embryonic stages at mRNA level (Additional file 1: Fig. S1B-E, F, H). The initially observed outside-in appendage phenotype was validated for both genes using two independent, non-overlapping fragments (NOFs) (Fig. 1c, d).

### The “*flipflop* syndrome”

RNAi mediated knockdown of both *Tc-ff* genes results in the outside-in appendage phenotype but also reveals additional lesions of the larval cuticle such as the invagination of abdominal segments and the failure of dorsal closure. These alterations from the wildtype collectively comprise the “*flipflop* syndrome”.

**Fig. 3:**
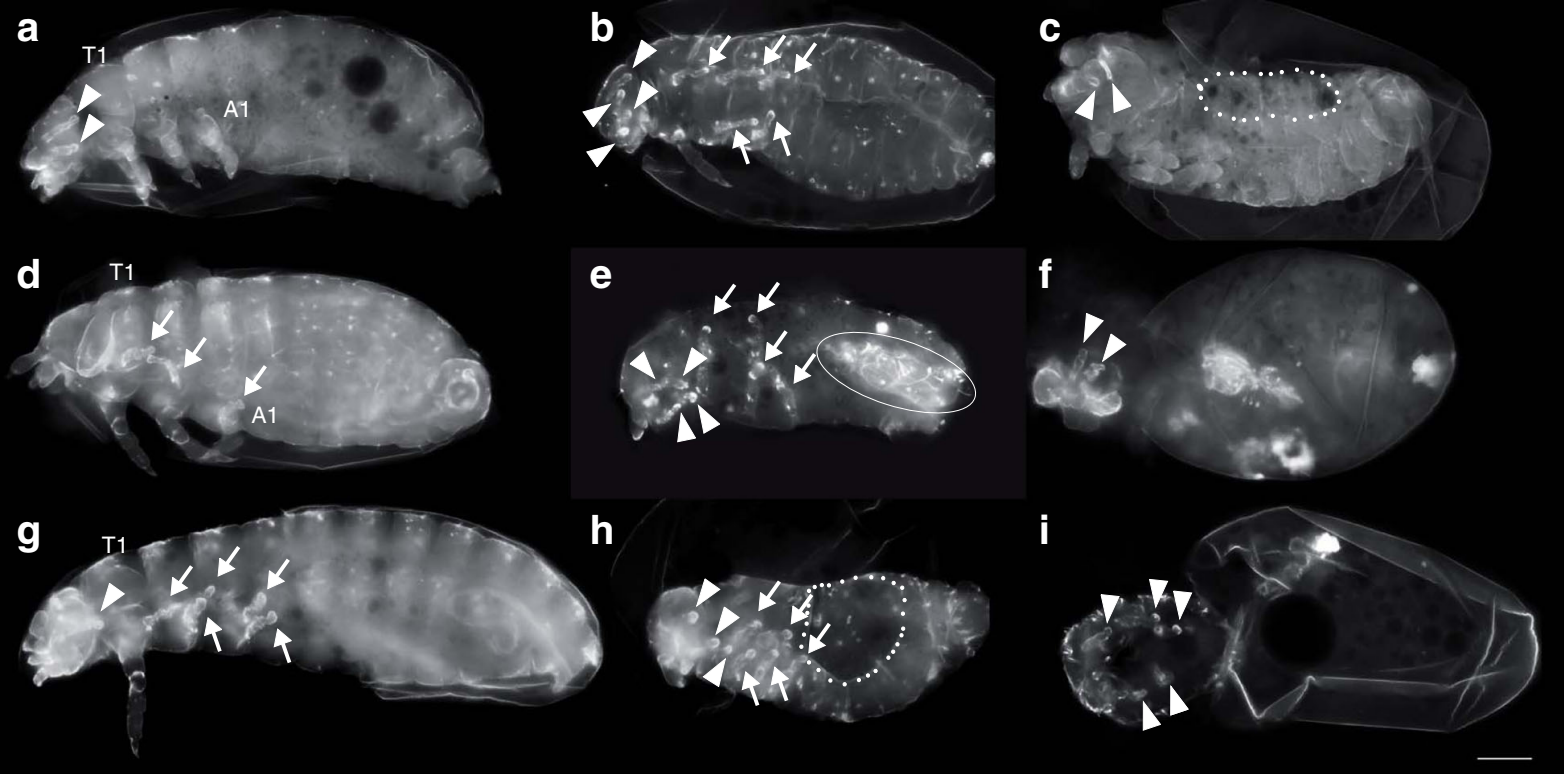
The “*flipflop* syndrome”. **a**-**i** Larval RNAi phenotypes of *Tc-ff1* **a**-**c**, *Tc-ff2* **d**-**f** and *Tc-ff1/ff2* **g**-**i** parental double-knockdown. **a**-**i** The enigmatic phenotype of inverted head appendages (arrowhead) and legs (arrows) in different combinations. In addition, inverted abdominal segments (**e**, circle) and dorsal openings (**c**, **h**, dotted line) are detected. **f**, **i** In stronger cuticle phenotypes cuticle remnants also show inverted appendages. A1 abdominal segment 1; T1 thoracic segment 1; scale bar 100 μm; all panels in all pictures: anterior to the left

**Fig. 4:**
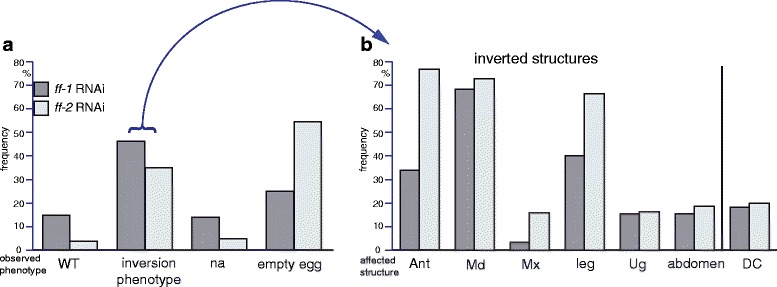
Quantitative analysis of *Tc-ff1* and *Tc-ff2* phenotypes **a** Classification of cuticle phenotypes into wildtype-like (WT), analysable cuticles with inversions, not analysable cuticles (na) and “empty eggs” without visible cuticle within the vitelline membrane. **b** Detailed description of the “Analysable cuticles”-class (Arrow from A to B) subdivided into inversion events of antenna (Ant), mandible (Md), maxilla (Mx), leg, urogomphi (Ug) and abdominal segments as well as cuticles with a dorsal opening (DC). Ant antenna; DC dorsal closure; Md mandible; Mx maxilla; na not analysable; Ug urogomphi; WT wildtype

In addition to the phenotype of inwards grown head appendages and legs, the invagination phenotype either included posterior abdominal segments (*ff1*, 16%, *n* = 205; *ff2*, 19%, *n* = 113) (Fig. 3e) or just the appendages of the last abdominal segment (urogomphi) (*ff1*, 16%, *n* = 205; *ff2*, 17% *n* = 113). Only stronger affected cuticles display an incomplete closure of the dorsal epidermis (*ff1*, 19%, *n* = 205; *ff2*, 20%, *n* = 113) (Fig. 3c, h). Not analysable cuticle remnants (*ff1*, 14%, *n* = 205; *ff2*, 5%, *n* = 113) (Fig. 3f) and the complete failure of cuticle formation (“empty egg”) (*ff1*, 25%, *N* = 443; *ff2*, 55%, *N* = 322) represented the strongest detectable knockdown effects. RNAi experiments for either *Tc-ff1* or *Tc-ff2* displayed the whole range of the “*flipflop* syndrome” but differed in penetrance being higher in *Tc-ff2* RNAi compared to *Tc-ff1* (Fig. 4). The phenotype of inwards grown head appendages and legs was observed more often in *Tc-ff2* RNAi experiments (67%, *n* = 113) when compared to *Tc-ff1* (*ff1*, 40%, *n* = 205) (Fig. 4). The number of inversion events observed varies from only one inverted appendage (see for example Fig. 1b´) to as many as 8 and 9 (Fig. 3b–h) or even 10 (not shown) in more extreme cases (Additional file 2: Table S2). Combined RNAi experiments that included double stranded RNA for both genes did not result in stronger or different cuticle phenotypes compared to the single RNAi experiments (Additional file 2: Table S1), nor were “empty egg” phenotypes more frequent. The quantitative analysis was carried out for the first two egg lays of RNAi experiments using a NOF directed at the 3′ region of the genes (Fig. 1c). We were able to validate the *flipflop* outside-in phenotype using the 5′ NOF but with a much lower frequency as the majority of embryos did not reach the larval stage (“empty egg”). Using antisense mRNA probes for both genes on *Tc-ff* ^RNAi^ embryos we observed strongly reduced staining compared to untreated wildtype embryos (Additional file 1: Fig. S1F-H), indicating the efficiency of the knockdown.

### *Tc-flipflop* determines the directionality of embryonic appendage formation in *Tribolium*

**Fig. 5:**
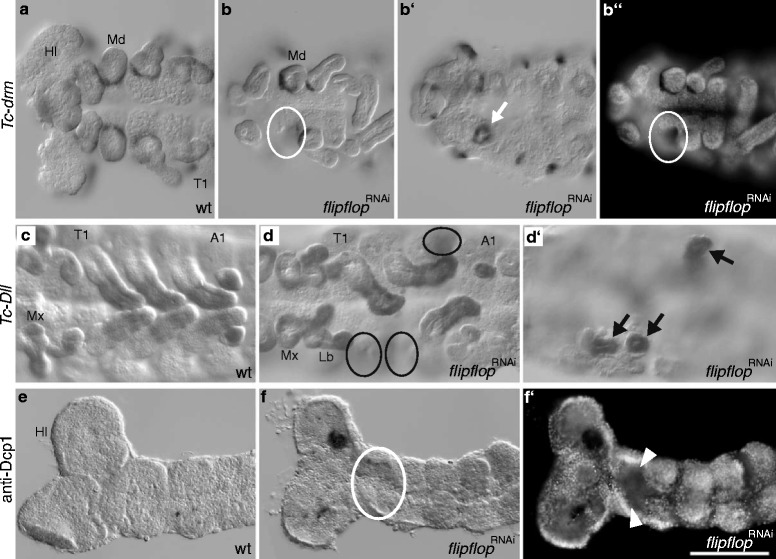
Marker gene expression and apoptosis analysis in *Tc-ff* ^RNAi^ embryos. Ventral focal plane: a, b, b´´, b, d, e-f´. dorsal focal plane: b´, d´. **a**-**b´´**: *Tc-drumstick*; **c**-**d´**: *Tc-Distalless*; **e**-**f´**: apoptosis-marker anti-Dcp1. (**a**, **b**-**b″**) *Tc-drumstick* (*Tc-drm*; TC006347) marker gene expression in embryonic wildtype (WT) and *Tc-ff* ^RNAi^ embryos marks the segmental base of appendages. (**b**, **b′**) Inverted mandible clearly displays wildtype *Tc-drm* expression at the dorsal focal plane (**b′**). (**c**, **d, d’**) *Tc-Distalless* (*Tc-Dll*) marker gene expression in embryonic wildtype (**c**) and *Tc-ff* ^RNAi^ embryos (**d, d’**) marks the distal portion of each appendage except for the mandible. (**d**) Some leg anlagen are not visible in the ventral focal plane (circles) but are visible as inverted limb buds in the dorsal focal plane (arrows) (**d’**). (**e**-**f**) Apoptosis marker anti-Dcp1 in wildtype (**e**) and *Tc-ff* ^RNAi^ embryos (**f**-**f′**). (**d**) Inverted appendage buds do not show elevated cell death at the stage of bud formation (circles). (**f′**) Nuclear staining (DAPI) visualises embryonic morphology of inverted appendages after *Tc-ff* knockdown. A1 abdominal segment 1; Lb labium; Md mandible; Mx maxilla; T1 thoracic segment 1; scale bar 100 μm; all panels in all pictures: anterior to the left

To answer the question of when during development of *Tc-ff* ^RNAi^ embryos the invagination of appendages starts, we analysed the early embryonic morphology as well as the expression profile of the distal leg marker *Tc-Distalless* (*Tc-Dll*) [4, 8] and the segmentally expressed *Tc-drumstick* (*Tc-drm, odd-skipped* family) [16] as a marker for proximal appendage border. We found that eversion defects occur already at the beginning of appendage bud formation (Fig. 5f‘), becoming clearly visible in elongated buds. When focusing on the ventral side of the germ band not all limb buds are visible (Fig. 5d, circles). The missing limbs can be detected, however, as invaginated appendages at a deeper focal plane (Fig. 5d´, arrows). Appendage marker gene analyses for *Tc-drm* (Fig. 5a-b”) and *Tc-Dll* (Fig. 5c-d‘) revealed that inverted appendages display wildtype expression patterns, confirming this phenotype is due to defects in growth directionality but not faulty pattern formation. As a result, embryonic inverted appendages displayed the wildtype-specific expression pattern of both, *Tc-drm* expression at the segmental base (Fig. 5b‘) and *Tc-Dll* expression in the distal region (Fig. 5d‘).

It has been proposed that epithelial morphogenesis can depend on an elevated level of cell death [32]. To reveal whether this may play a role in the directionality of appendage growth in *Tribolium*, we analysed *Tc-ff* ^RNAi^ embryos using an antibody detecting Dcp1 [42]. However, the ingression of cells does not appear to be accompanied by an elevated level of cell death in the limb primordium or its immediate neighbourhood (Fig. 5f).

### Knockdown of the highly-conserved *Tc-RhoGEF2* gene displays a *Tc-flipflop*-like RNAi phenotype

**Fig. 6:**
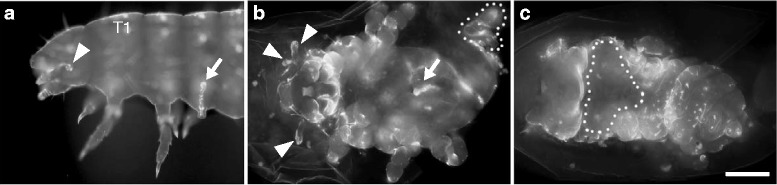
Larval *Tc-RhoGEF2* ^RNAi^ phenotype. **a** Magnified view of the KT221 mutant phenotype displaying an inverted leg (arrow) and head appendage (arrowhead). **b**, **c** *RhoGEF2* ^RNAi^ cuticles display more severe defects: in addition to inverted appendages of the head (arrowheads) and thorax (arrows) *RhoGEF2* knockdown also results in segmentation defects, a malformed posterior abdomen (**b** dotted outline) and dorsal closure defects (**c**, outlined with dots). Extended focus in all pictures combines several optical sections. **a** lateral view; **b** ventral view; **c** dorsal view); **b**, **c** scale bar 100 μm

To find additional putative interaction partners of *Tc-ff*, we searched the iBeetle database [10] for the characteristic *Tc-ff*-like outside-in RNAi phenotype. This approach follows the logic that a similar phenotype indicates the involvement in a similar pathway. Indeed, appendage eversion was disrupted when a gene coding for a Rho-GTP exchange factor was knocked down via RNAi. The analysis of the genomic region within the *Tribolium* genome revealed a gene model (au2.g3948.t2) that combines two adjacent models from previous annotations (TC003069 and TC003070). BLAST analysis identifies this gene as the *Drosophila* homolog of the highly-conserved Rho Guanine nucleotide Exchange Factor 2 in *Tribolium* (*Tc-*RhoGEF2). The lack of sequence conservation of the *Tc-ff* orphan genes made it difficult to relate their function to known cellular pathways. Fortunately, after the discovery of *Tc-ff1*, another outside-in leg phenotype was found during an investigation of lethal mutants identified in the GEKU transposon mutagenesis screen [55]: the insertion line KT221 produced homozygous embryos which occationally display outside-in appendages. The KT221 insertion is located in an intron of the *Tc-RhoGEF2* gene identified via the iBeetle database. RNAi mediated knockdown of *Tc-RhoGEF2* generally results in much more severely affected larval cuticles that often are hard to analyse. However, *Tc-RhoGEF2* ^RNAi^ cuticles (*N* = 138) do reveal the characteristic *Tc-ff*-like phenotype of inverted appendages (29%) and incomplete dorsal closure (58%) (Fig. 6).

### *Tc-flipflop* and *Tc-RhoGEF2* influence morphogenetic dynamics and polarity of extraembryonic membranes and embryonic tissues

To determine the function of *Tc-ff* and *Tc-RhoGEF2* in early embryogenesis we analysed RNAi embryos both via time-lapse microscopy and in fixed stages. We found that RNAi mediated knockdown of *Tc-ff* as well as *Tc-RhoGEF2* influences the integrity of extraembryonic membranes and interferes with morphogenetic movements of the young germ anlage and the germ band.

**Fig. 7:**
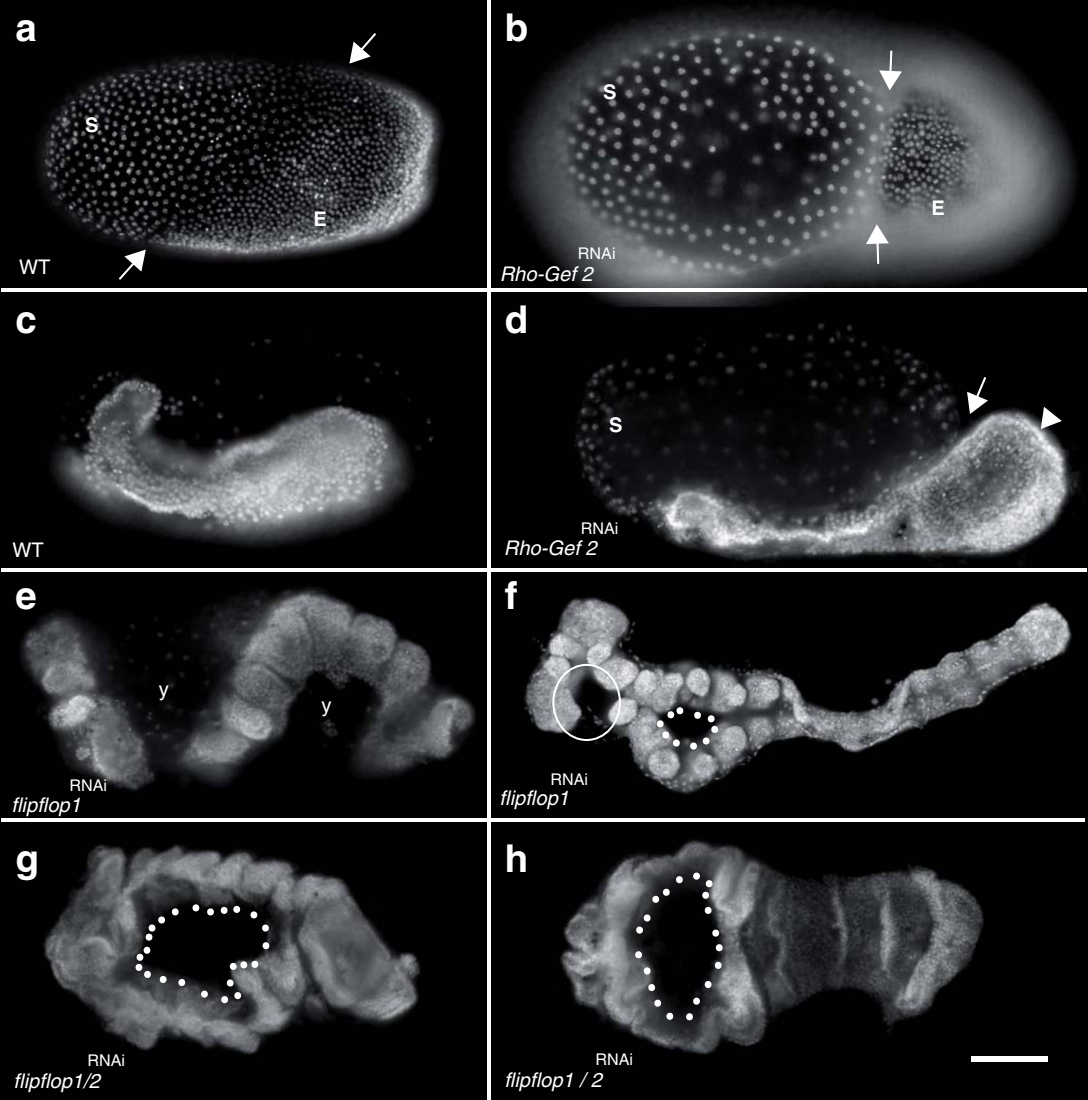
*flipflop*- and *RhoGEF2*-RNAi knockdown interferes with early embryonic morphogenesis. **a** Wildtype blastoderm stage displays distinction between anterior extraembryonic tissue-anlagen (serosa S) and the posterior positioned embryonic cells (E); arrows mark the border. **b** Fixed RNAi embryos reveal tissue constrictions in blastoderm stages during embryonic anlagen formation (arrows). **c** Wildtype embryo prior to axis elongation. **d** Young *RhoGEF2* ^RNAi^ embryo with thickened posterior growth zone (arrowhead). The affected embryo is excluded from the serosa (arrow). **e**, **h** Extended germband displays an unusual S-like orientation within the yolk (y). **e**, inverted head appendages (**f**, circle) appear missing when viewed ventrally. **f, g, h** Median holes in *ff1*- and *ff1/RhoGEF2* ^RNAi^ embryos (dotted line). **a**, **c**, **d**, **e** Lateral view; **f**-**g** ventral view; scale bar 100 μm; all panels in all pictures: anterior to the left; Nuclear DAPI staining

In late blastoderm stages we observed deep constrictions between the extraembryonic membrane and the site of the presumptive embryonic cells (Fig. 7b). Other embryos were able to form a germ band that is excluded from the serosa and displays a thickened posterior growth zone (Fig. 7d). Moreover, we frequently observed that germband extension underwent an S-shaped progression through the yolk (Fig. 7e). Such embryos often display holes along the ventral midline during older stages (Fig. 7g, h).

**Fig. 8:**
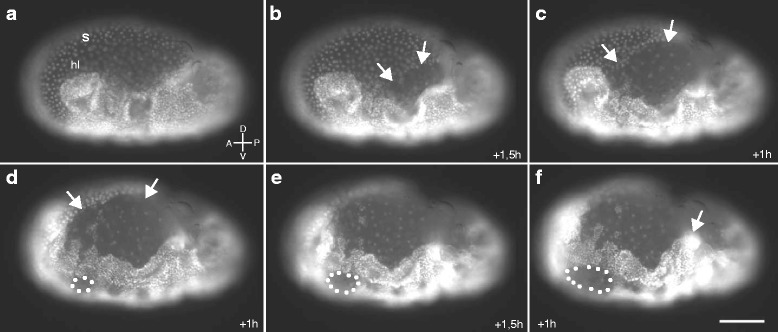
Live imaging stills of an *Tc-ff/RhoGEF2* ^*double-*RNAi^ embryo. **a**-**f** Developmental dynamics in a timeframe of 6 h, starting a few hours after egg lay. **a** Germ anlage covered by the intact serosa (S). **b**-**f** Rupture and successive retraction of extraembryonic membranes (arrows). **d**-**e** Holes form in the median regions of the embryo (dotted outline) and ingression of embryonic tissue into the yolk (arrow in f). **a**-**f** Lateral view; anterior to the left, ventral down; scale bar 100 μm

Via a live-imaging approach using a nGFP line [43] we observed that the extraembryonic membranes are functionally impaired. In the embryo shown in Fig. 8, the rupture and successive retraction of extraembryonic tissue during germ band elongation is visible while embryonic tissue develops holes and partly ingresses into the yolk.

## Discussion

Genes that are required for the allocation and patterning of appendages are well-known [2, 59]. However, not much is known about the factors that determine the initial direction of tissue evagination during appendage bud formation. Here, we show that the novel genes *Tc-ff1* and *Tc-ff2* are required for early appendage eversion during embryogenesis of *Tribolium*. Knockdown of those genes leads to an outside-in phenotype of inverted appendages that has not been described so far. We further observed this highly specific appendage inversion phenotype after knockdown of *RhoGEF2* function. This leads us to the hypothesis that the *Tc-ff* genes serve as important co-regulators within a Rho-dependent pathway.

### Eversion of embryonic limb anlagen requires the novel *flipflop* genes in *Tribolium*

In the wildtype *Tribolium* embryo, limb development starts as a bud that everts and subsequently grows in length. For the first time, we identified genetic components required for this important initial decision within the limb field tissue. Initially, *Tc-ff1* was uncovered via its enigmatic inverted leg phenotype during the iBeetle RNAi pre-screen using randomly picked cDNA clones as a source for dsRNA [48]. By sequence homology we identified a second *flipflop* gene in the *Tribolium* genome, named *Tc-ff2*. Both genes are predicted to code for small proteins of 136 and 127 amino acids, respectively, lacking any known functional domains. A web-tool based analysis of their sequences [1, 26] characterises the *Tc-ff* genes, at least in the absence of a binding partner, as “unstructured” with long stretches of low complexity domains.

To answer the question, whether the *Tc-ff* genes are indeed protein-coding or function as long-non-coding RNAs, we raised anti-peptide-antibodies that recognise a single specific band of the expected molecular weight (Additional file 1: Fig. S1A) in a Western blot using embryonic extracts. We take this as indication that the predicted ORFs are indeed translated, however, the antibody does not detect a distinct spatial or subcellular pattern in the embryo.

The *Tc-ff* genes can be found in the *Tribolium* lineage (including *T. confusum*, *T. madens* and *T. freemani*. UCSC Genome Browser) but seem absent from any other sequenced genomes. Based on their lack of conservation, small size and apparent disordered structure, we classify *Tc-ff1* and *Tc-ff2* as orphan genes [53]. Given that orphan genes of small size tend to be evolutionarily more recent [31], these genes may well be limited to a small subset of colepteran species. However, we also cannot exclude that the *Tc-ff* genes provide a conserved function and homologs cannot be identified due to small size and a fast-evolving sequence. In any case, the *Tc-ff* genes represent one of few examples of orphan genes with an essential early embryonic function. In addition, these genes illuminate an early decision in the development of appendages which had been overlooked so far.

With the *Tc-ff* knockdown we observed a variety of phenotypes in affected larval cuticles ranging from severe to weak. We categorise the inverted growth of one or just a few appendages without additional defects as a weak knockdown phenotype. More severely affected cuticles display many outside-in events at once combined with a dorsal closure defect (Fig. 3h). The strongest effects are represented by cuticle remnants without recognisable structures or the complete lack of a cuticle (“empty eggs”), respectively, underlining that *Tc-ff* function is required in different tissues. While the *Tc-ff2* knockdown results in a somewhat higher penetrance regarding phenotypic defects compared to *Tc-ff1*, both genes appear to be non-redundant as the single knockdown of either gene is sufficient to disrupt their morphogenetic function. This is also highlighted by the fact that the combined *Tc-ff1/ff2* double RNAi knockdown does not increase the overall penetrance compared to the single knockdowns (Additional file 2: Table S1).

We found that the direction of limb budding in *Tribolium* is determined as early as the beginning of appendage bud formation and is significantly influenced by *Tc-ff* function. We have no indications that in *Tribolium* the eversion of the limb epithelium depends on an elevated level of cell death as it has been shown for other cases [32, 44].

### The *flipflop* genes may act in a RhoGEF-dependent cell polarity network

Given that the orphan *Tc-ff* genes lack evolutionarily conserved characteristics to associate them with any known molecular pathway, we searched for other genes that display the same or a similar RNAi knockdown phenotype. We found that RNAi-mediated (partial) knockdown of the ubiquitously expressed *Tribolium* homolog of *RhoGEF2* did also result in the disruption of appendage eversion, resembling the characteristic *Tc-ff* RNAi outside-in phenotype (Fig. 6). In contrast to *Tc-ff*, *Tc-RhoGEF2* represents an evolutionarily highly conserved gene. By activating members of the Rho-family GTPase [3], RhoGEF2 plays an essential role in a number of morphological processes involving cell-cell adhesion, cell polarity, cell migration and cell motility [20, 36, 47]. Furthermore, RhoGEF2 is well known to be a key factor controlling cell shape change and apical constriction through the regulation of actomyosin contractility [3, 15, 24, 38]. In *Drosophila* an impairment of factors involved in Rho-dependent apical constriction can result in salivary gland formation outside the embryo, instead of forming inside the embryo as in wildtype [7]. Here, we show that both *Tc-ff* and *Tc-RhoGEF2* initially determine the direction of appendage growth. However, the number and localisation of inside-out events seen in different *Tc-ff* ^RNAi^ larvae does not follow a certain pattern. This variability of the phenotype led us to hypothesise that the cell fate decision of the epithelium whether to invaginate or evaginate may be a quantitative local event rather than an all-or-nothing epithelial switch. Depending on the number of cells within the limb field undergoing uniform polarised constriction either at the apical or the basal side provides the physical ground for an epithelium to buckle to one of the two directions. Disruption of the uniformity of this collective cell behaviour through absence of the same polarity cue in all cells increases the likelihood of adjacent cells being forced into a different direction. In *Drosophila* it has been shown that inhibition of apical constriction in a defined area of the epithelial tissue disrupts ventral furrow formation depending on the number of cells affected as well as the intensity of the inhibitory signal [13]. Tissue invagination does still function when a smaller section of cells is affected as long as there are still enough cells undergoing cell shape change “dragging” adjacent corrupted cells with them.

Based on the findings in *Drosophila* and the highly conserved RhoGEF2 function, we hypothesise that in *Tribolium ff* and *RhoGEF2* play an essential role in the decision where cellular constriction - either at the apical or basal side - takes place, so that in the absence of this apical-basal polarity cue the buckling direction becomes random. However, the genes are not required for the formation of the appendage primordium itself. Thus, we propose that *Tc-ff* is involved in a Rho GTPase-dependent pathway that regulates the apico-basal polarity of a cell. However, a detailed analysis of the cellular dynamics including suitable markers localising proteins that are involved in cell shape change events will be required to validate our hypothesis.

### *Tc-flipflop* and *Tc-RhoGEF2* are also required for the morphogenetic dynamics and polarity of extraembryonic membranes

In contrast to the single reduced amnioserosa of *Drosophila*, *Tribolium* has two extraembryonic membranes, amnion and serosa, that actively contribute to the morphogenesis of *Tribolium* during gastrulation, germ band extension and dorsal closure [18, 39]. We have seen that knockdown of either *Tc-ff* or *Tc-RhoGEF2* affects the directed morphogenetic movements and the cellular integrity of extraembryonic tissues. This aspect of *Tc-ff* and *Tc-RhoGEF2* function is seen in the early embryo where the extraembryonic membranes fold prematurely and fail to fully cover the embryo. As a consequence, a misshaped germband forms on top of the yolk (Fig. 7d). This phenotype clearly illustrates that the dorso-posterior translocation of the extraembryonic tissues and the enwrapping of the embryo require coordinated tissue elongation within a plane of cells, a process likely to involve planar cell polarity (PCP) [57]. As described for other systems, Rho-dependent cell shape changes can be regulated through the PCP pathway that involves non-canonical Wnt signalling. Thus, we propose that the polarised maintenance of extraembryonic tissue dynamics during *Tribolium* embryogenesis depends on PCP and that *Tc-RhoGEF2* and the *Tc-ff* genes are downstream targets of this important signalling pathway in *Tribolium*.

Additionally, we have observed premature ruptures of extraembryonic membranes in RNAi embryos (Fig. 8) as well as dorsal closure defects. In *Drosophila*, dorsal closure depends on actomyosin contractility at the apical cortex of the amnioserosa cells while the actin cable in cells of the leading edge seems dispensable for this process [11, 40]. It is conceivable that the polarity of the extraembryonic cells along the apico-basal axis might also be disturbed in *Tc-ff* ^RNAi^ embryos and, as a consequence, dorsal closure is impaired. An unstructured actomyosin network also may contribute to weaken the integrity of the extraembryonic membranes epithelia so that they cannot withstand strong mechanical tension during the dorsal closure process. Once cell polarity markers become available for *Tribolium* it will be feasible to evaluate the involvement of PCP in the process of dorsal closure.

Based on our observations we hypothesise that *Tc-ff* and *Tc-RhoGEF2* contribute to the polarisation of cell movement early in development but do not influence the differentiation of epithelial structures per se. Later, they are involved in stabilising the cellular components required for the pulling forces of tissues during dorsal closure.

Assuming that the *Tc-ff* genes act in the same pathways as *Tc-RhoGEF2*, we propose that they may be downstream targets of non-canonical Wnt/PCP signalling via the regulation of or in parallel to Rho proteins.

## Conclusions

Here, we showed *Tc-ff* as one of the few examples of an orphan gene playing a crucial role in a developmental process such as in morphogenetic cell movements. This is one more example for an additional novel, species-specific or fast evolving factor that functions in an otherwise conserved pathway. Previously, an orphan gene within the BMP-pathway involved in digit formation and –outgrowth in the limb has been described in a vertebrate [23]. It will be interesting to see if functional equivalents of the *Tc-ff* orphan genes will be found in other organisms.

## Additional files

Additional file 1: Figure S1. Flipflop expression analysis. (**A**) Western blots mark specific bands presumably representing the Flipflop proteins according to the expected molecular weight (FF1 14,47 kD; FF2 13,63 kD, asterisks). Unspecific bands of higher molecular weight may detect yolk proteins. (**B**-**E**) whole mount in situ hybridisation using *Tc-flipflop1* and *Tc-flipflop2* mRNA antisense-probes displays ubiquitous expression patterns exemplarily shown in wildtype (wt) embryos both before and at the stage of bud formation. (**F**-**I′**) Transcript detection in wildtype embryos (**F**, **F′**, **H**, **H′**) and after knockdown of the respective gene (**G**, **G’**, **I**, **I′**) to validate knockdown efficiency. (**F′**, **G’**, **H′**, **I′**) DAPI staining. Scale bar 100 μm; all panels in all pictures: anterior to the left. Western blot analysis. (PDF 150 kb)

Additional file 2: Table S1. Quantitative analysis of phenotypes. **Table S2.** Count of analysable inversion events per cuticle. **Table S3.** Quantitative analysis of the wildtype and “empty egg” phenotype. (PDF 32 kb)
