# Supplementary material for: The flipflop orphan genes are required for limb bud eversion in the Tribolium embryo
Source: Front Zool. 2017 Oct 19;14:48. doi: 10.1186/s12983-017-0234-9 (PMC5649079; doi:10.1186/s12983-017-0234-9)
Supplement: Supplementary file 1 — Flipflop expression analysis. (A) Western blots mark specific bands presumably representing the Flipflop proteins according to the expected molecular weight (FF1 14,47 kD; FF2 13,63 kD, asterisks). Unspecific bands of higher molecular weight may detect yolk proteins. (B-E) whole mount in situ hybridisation using Tc-flipflop1 and Tc-flipflop2 mRNA antisense-probes displays ubiquitous expression patterns exemplarily shown in wildtype (wt) embryos both before and at the stage of bud formation. (F-I′) Transcript detection in wildtype embryos (F, F′, H, H′) and after knockdown of the respective gene (G, G’, I, I′) to validate knockdown efficiency. (F′, G’, H′, I′) DAPI staining. Scale bar 100 μm; all panels in all pictures: anterior to the left. Western blot analysis. (PDF 150 kb) [file 12983_2017_234_MOESM1_ESM.pdf]

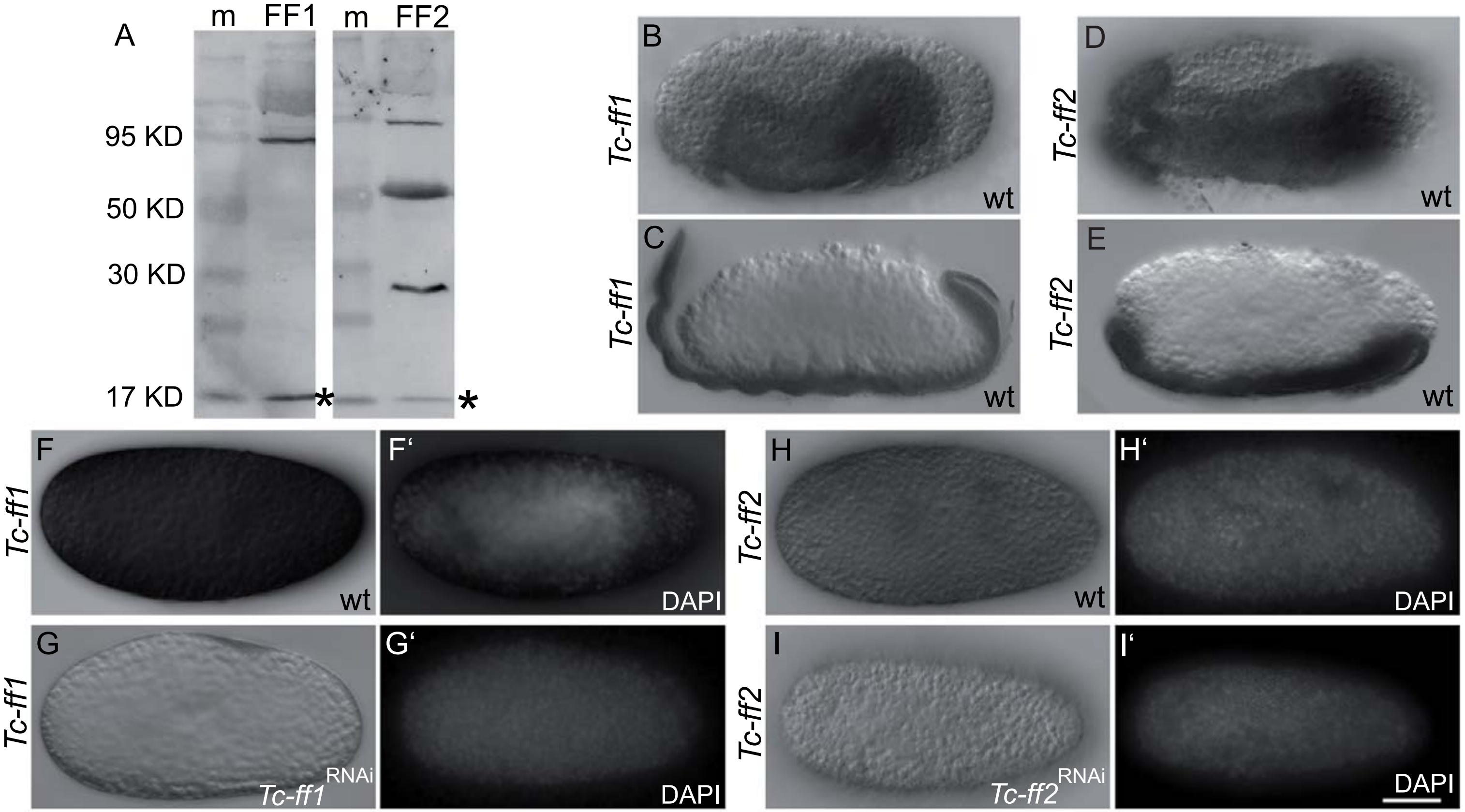
A
m
FF1
m
FF2
95 KD
50 KD
30 KD
17 KD
*
*
B
Tc-ff1
wt
C
Tc-ff1
wt
D
Tc-ff2
wt
E
Tc-ff2
wt
F
Tc-ff1
wt
F‘
DAPI
G
Tc-ff1
Tc-ff1 RNAi
G‘
DAPI
H
Tc-ff2
wt
H‘
DAPI
I
Tc-ff2
Tc-ff2 RNAi
I‘
DAPI

Western blot analysis

For protein detection protein suspensions of *Tribolium* embryos of different developmental stages were produced. Embryos were bleached, washed and mechanically solubilised in Breaking buffer. After centrifugation, the supernatant was run on a 12% polyacrylamide gel and blotted overnight. Membranes were blocked, washed and incubated overnight using antibodies raised against Flipflop1 and Flipflop2 peptides (see Materials and Methods). Incubation using a secondary antibody (Biotin-SP-conjugated AffiniPure Anti-Rabbit IgG, Jackson ImmunoResearch Europe Ltd) was carried out for an hour at room temperature. Vectastain ABC-AP (Vector Laboratories) and NBT/BCIP (Roche Applied Science) were used for detection.
