# Supplementary material for: The flipflop orphan genes are required for limb bud eversion in the Tribolium embryo
Source: Front Zool. 2017 Oct 19;14:48. doi: 10.1186/s12983-017-0234-9 (PMC5649079; doi:10.1186/s12983-017-0234-9)
Supplement: Supplementary file 2 — Quantitative analysis of phenotypes. Table S2. Count of analysable inversion events per cuticle. Table S3. Quantitative analysis of the wildtype and “empty egg” phenotype. (PDF 32 kb) [file 12983_2017_234_MOESM2_ESM.pdf]

**Supplementary Material** **Thümecke et al.**

**Table S1 Statistical analysis of phenotypes**

| | Ant | Md | Mx | leg | Ug | abdomen | DC |
|---|---|---|---|---|---|---|---|
| Tc-ff1 (n=205) | 34% | 69% | 3% | 40% | 16% | 16% | 19% |
| Tc-ff2 (n=113) | 77% | 73% | 16% | 67% | 17% | 19% | 20% |
| Tc-ff1/ff2 (n=113) | 57% | 70% | 9% | 43% | 27% | 19% | 35% |

Statistical analysis of affected appendages in single- and double *flipflop* RNAi experiments. Ant antenna; DC dorsal closure defect; Md mandible; Mx maxilla.

**Table S2 Number of inverted appendages per analysable cuticle**.

| | 1 | | 2 | | 3 | | 4 | | 5 | | 6 | | 7 | | 8 | | 9 | | 10 | |
|---|---|---|---|---|---|---|---|---|---|---|---|---|---|---|---|---|---|---|---|---|
| | % | n | % | n | % | n | % | n | % | n | % | n | % | n | % | n | % | n | % | n |
| Tc-ff1 | 27 | 43 | 25 | 40 | 21 | 33 | 11 | 18 | 4 | 7 | 6 | 9 | 3 | 4 | 1 | 1 | 1 | 1 | 2 | 3 |
| Tc-ff2 | 28 | 14 | 16 | 12 | 14 | 11 | 14 | 11 | 10 | 8 | 10 | 8 | 6 | 5 | 4 | 3 | 4 | 3 | 3 | 2 |
| Tc-ff1/ff2 | 17 | 16 | 17 | 16 | 19 | 18 | 22 | 21 | 10 | 9 | 7 | 7 | 3 | 3 | 3 | 3 | 0 | 0 | 1 | 1 |

Only clearly identified appendages include legs, mandibles, antennae and urogomphi have been taken into this analysis.

**Table S3 Statistical quantification of the wildtype and "empty egg" phenotype**

| | WT | empty egg |
|---|---|---|
| Tc-ff1 (n=443) | 15% | 25% |
| Tc-ff2 (n=322) | 4% | 55% |
| Tc-ff1/ff2 (n=287) | 3% | 43% |
